# Supplementary material for: MetaRibo-Seq measures translation in microbiomes
Source: Nat Commun. 2020 Jun 29;11:3268. doi: 10.1038/s41467-020-17081-z (PMC7324362; doi:10.1038/s41467-020-17081-z)
Supplement: Supplementary file 10 — Supplementary Data 7 [file 41467_2020_17081_MOESM10_ESM.zip › File2/Confidence_High_Taxonomy/107689_out.krona.html]

Javascript must be enabled to view this page.

members
magnitude
magnitudeUnassigned
count
unassigned
taxon
rank

107689\_out

179

179
superkingdom
2

179
1239
phylum

179
class
186801

order
186802
179

186806
family
1

genus
1730
1


SRS144506\_contig\_number\_2899
species
39496
1

3

SRS017191\_contig\_number\_contig-100\_25737.70455SRS049446\_contig\_number\_contig-100\_35967.35968SRS893230\_contig\_number\_contig-100\_25657.25658
family
541000
178

species

SRS011084\_contig\_number\_contig-100\_1952.180951SRS011302\_contig\_number\_24020SRS011405\_contig\_number\_7285SRS012273\_contig\_number\_contig-100\_4010.230962SRS012969\_contig\_number\_25143SRS013098\_contig\_number\_contig-100\_2649.151296SRS013158\_contig\_number\_contig-100\_5004.5004SRS013476\_contig\_number\_contig-100\_1804.68343SRS013638\_contig\_number\_14623SRS013940\_contig\_number\_contig-100\_758.122920SRS013951\_contig\_number\_28993SRS013965\_contig\_number\_39416SRS014235\_contig\_number\_contig-100\_14246.178096SRS014459\_contig\_number\_contig-100\_4215.180193SRS014613\_contig\_number\_contig-100\_10523.81604SRS014736\_contig\_number\_contig-100\_9666.9667SRS014855\_contig\_number\_5198SRS014923\_contig\_number\_42743SRS014948\_contig\_number\_15470SRS014979\_contig\_number\_33814SRS015065\_contig\_number\_contig-100\_4497.179499SRS015190\_contig\_number\_contig-100\_9131.77348SRS015264\_contig\_number\_contig-100\_4758.4758SRS015431\_contig\_number\_37949SRS015579\_contig\_number\_25494SRS015663\_contig\_number\_32036SRS015782\_contig\_number\_39995SRS015854\_contig\_number\_19075SRS016018\_contig\_number\_14604SRS016335\_contig\_number\_contig-100\_7333.152347SRS016954\_contig\_number\_contig-100\_6701.102708SRS017103\_contig\_number\_31933SRS017521\_contig\_number\_46516SRS018313\_contig\_number\_contig-100\_8435.71194SRS018351\_contig\_number\_1380SRS018427\_contig\_number\_contig-100\_18179.61117SRS018623\_contig\_number\_11519SRS018656\_contig\_number\_contig-100\_241.76664SRS018817\_contig\_number\_21498SRS018836\_contig\_number\_1096SRS018984\_contig\_number\_14006SRS019068\_contig\_number\_38758SRS019161\_contig\_number\_29718SRS019445\_contig\_number\_contig-100\_4868.142447SRS019968\_contig\_number\_11920SRS020233\_contig\_number\_44201SRS020328\_contig\_number\_contig-100\_2320.186644SRS021484\_contig\_number\_17412SRS022071\_contig\_number\_contig-100\_3312.188302SRS022137\_contig\_number\_contig-100\_4175.83437SRS022524\_contig\_number\_contig-100\_2006.76263SRS023971\_contig\_number\_contig-100\_2216.2216SRS024009\_contig\_number\_8651SRS024132\_contig\_number\_contig-100\_9471.214047SRS024331\_contig\_number\_22452SRS042628\_contig\_number\_contig-100\_7482.157804SRS042966\_contig\_number\_contig-100\_2240.114298SRS043001\_contig\_number\_3312SRS043411\_contig\_number\_contig-100\_1279.104478SRS043768\_contig\_number\_contig-100\_9923.143846SRS043841\_contig\_number\_contig-100\_17620.17621SRS045739\_contig\_number\_contig-100\_526.526SRS045826\_contig\_number\_contig-100\_30325.30326SRS046502\_contig\_number\_9348SRS047014\_contig\_number\_11790SRS049402\_contig\_number\_contig-100\_2522.103137SRS049995\_contig\_number\_28648SRS050422\_contig\_number\_contig-100\_11129.82733SRS050925\_contig\_number\_contig-100\_8513.136720SRS051031\_contig\_number\_15559SRS051610\_contig\_number\_contig-100\_1685.59512SRS051882\_contig\_number\_contig-100\_3954.97174SRS052697\_contig\_number\_32751SRS053214\_contig\_number\_contig-100\_3028.99246SRS054905\_contig\_number\_4935SRS054956\_contig\_number\_14758SRS055982\_contig\_number\_contig-100\_3249.76170SRS056273\_contig\_number\_13812SRS057480\_contig\_number\_contig-100\_3712.3713SRS058770\_contig\_number\_26237SRS062654\_contig\_number\_contig-100\_13286.13287SRS063040\_contig\_number\_48317SRS063518\_contig\_number\_contig-100\_631.166720SRS063985\_contig\_number\_15456SRS064276\_contig\_number\_27973SRS064757\_contig\_number\_11477SRS075078\_contig\_number\_26793SRS075773\_contig\_number\_5899SRS075821\_contig\_number\_12580SRS075878\_contig\_number\_23929SRS075984\_contig\_number\_15697SRS076756\_contig\_number\_5643SRS077127\_contig\_number\_686SRS077194\_contig\_number\_19694SRS077231\_contig\_number\_18720SRS077294\_contig\_number\_contig-100\_689.117940SRS077392\_contig\_number\_40046SRS077641\_contig\_number\_17248SRS078242\_contig\_number\_contig-100\_5180.145832SRS097889\_contig\_number\_contig-100\_2007.200945SRS097920\_contig\_number\_28778SRS098073\_contig\_number\_contig-100\_23365.23366SRS098571\_contig\_number\_36385SRS098655\_contig\_number\_12120SRS098881\_contig\_number\_23424SRS101376\_contig\_number\_5007SRS101433\_contig\_number\_8829SRS104084\_contig\_number\_5503SRS1041031\_contig\_number\_20695SRS1041039\_contig\_number\_contig-100\_36312.73004SRS1041118\_contig\_number\_9491SRS1041129\_contig\_number\_contig-100\_160.89080SRS1041136\_contig\_number\_24617SRS1041137\_contig\_number\_19865SRS1041140\_contig\_number\_contig-100\_21338.57225SRS1041145\_contig\_number\_contig-100\_15294.49259SRS104636\_contig\_number\_contig-100\_2583.119773SRS104912\_contig\_number\_2872SRS104975\_contig\_number\_contig-100\_30476.30477SRS105153\_contig\_number\_36899SRS1055043\_contig\_number\_contig-100\_5622.132820SRS1055049\_contig\_number\_260SRS1055056\_contig\_number\_contig-100\_1612.73810SRS1055067\_contig\_number\_contig-100\_3762.77315SRS1055069\_contig\_number\_contig-100\_234.85075SRS1055076\_contig\_number\_contig-100\_13446.13447SRS1055099\_contig\_number\_contig-100\_5283.62530SRS140492\_contig\_number\_28275SRS142503\_contig\_number\_29491SRS142542\_contig\_number\_contig-100\_2743.142581SRS142890\_contig\_number\_contig-100\_530.251989SRS142923\_contig\_number\_934SRS143148\_contig\_number\_contig-100\_4024.113252SRS143417\_contig\_number\_contig-100\_2622.139708SRS143466\_contig\_number\_27501SRS143598\_contig\_number\_21179SRS143895\_contig\_number\_42193SRS143991\_contig\_number\_17661SRS144135\_contig\_number\_34026SRS144183\_contig\_number\_29821SRS144297\_contig\_number\_12662SRS144362\_contig\_number\_31021SRS144714\_contig\_number\_contig-100\_1389.135766SRS144753\_contig\_number\_3874SRS146812\_contig\_number\_43717SRS146813\_contig\_number\_26319SRS147022\_contig\_number\_contig-100\_20383.20383SRS147139\_contig\_number\_contig-100\_2430.191412SRS147346\_contig\_number\_47303SRS147377\_contig\_number\_1000SRS147557\_contig\_number\_23955SRS147653\_contig\_number\_6298SRS147919\_contig\_number\_12133SRS147977\_contig\_number\_1678SRS148159\_contig\_number\_48944SRS148196\_contig\_number\_27653SRS148253\_contig\_number\_contig-100\_2035.86431SRS148424\_contig\_number\_24294SRS148511\_contig\_number\_14819SRS148721\_contig\_number\_1117SRS148784\_contig\_number\_contig-100\_1577.139023SRS148817\_contig\_number\_contig-100\_1622.63314SRS149075\_contig\_number\_contig-100\_18344.59734SRS149325\_contig\_number\_contig-100\_21117.69894SRS149784\_contig\_number\_13181SRS149879\_contig\_number\_6358SRS892882\_contig\_number\_1190SRS893172\_contig\_number\_14003SRS893187\_contig\_number\_15000SRS893279\_contig\_number\_contig-100\_3581.73353SRS893366\_contig\_number\_contig-100\_6156.132860SRS893373\_contig\_number\_12649SRS893378\_contig\_number\_4980SRS971276\_contig\_number\_26394
1898205
174

1
genus
216851

species

SRS019030\_contig\_number\_14792
1946508
1
